# Yield Variation Characteristics of Red Paddy Soil under Long-Term Green Manure Cultivation and Its Influencing Factors

**DOI:** 10.3390/ijerph19052812

**Published:** 2022-02-28

**Authors:** Jun Xie, Feng Liang, Junjie Xie, Guanjie Jiang, Xinping Zhang, Qin Zhang

**Affiliations:** 1 Key Laboratory of Poyang Lake Watershed Agricultural Resources and Ecology of Jiangxi Province, Jiangxi Agricultural University, Nanchang 330045, China; xiejun09@outlook.com (J.X.); llangfeng1@126.com (F.L.); xiejunjie0129@163.com (J.X.); jiangguanjie@126.com (G.J.); zxp0523@outlook.com (X.Z.); 2 College of Land Resource and Environment, Jiangxi Agricultural University, Nanchang 330045, China

**Keywords:** long-term fertilizer, green manure, paddy soil, yield

## Abstract

Rice is an important food crop in China, fertilization measures significantly affect soil properties and ultimately change rice yield. Thus, examining the effects of long-term green manure cultivation on the rice yield and the driving factors on rice yield, plays a crucial role in maintaining food security. Based on the long-term green manure cultivation, the treatments included no fertilizer (CK), chemical fertilizer (NPK), chemical fertilizer + Chinese milk vetch (NPK + GM), chemical fertilizer + Chinese milk vetch + rice straws (NPK + GM + S), and chemical fertilizer + Chinese milk vetch + pig manure (NPK + GM + M) treatments. One-way repeated ANOVA was used to determine the effects of diverse fertilizer modes on temporal variations in rice yields. The redundancy analysis (RDA) was used to calculate the magnitudes of the effects of soil properties on rice yield. Compared with the CK treatment, four fertilizer treatments led to significantly increased double-season rice yields (116.40–124.49%), with no significant difference between four fertilizer treatments (*p* *>* 0.05). There were five soil properties accounting for 66.3% variation in rice yield (*p* *<* 0.05), with available potassium (AK) being the most influential factor (32.2% variation), whereas potential of hydrogen (pH), total nitrogen (TN), total phosphorus (TP), and soil organic carbon (SOC) accounted for 15.3%, 10.5%, 5.1%, and 3.2% variation in rice yield (*p* *<* 0.05), respectively. Thus, SOC, TN, TP, AK, and pH were major factors affecting the double-season rice yield of red paddy soil under long-term green manure cultivation. However, the results suggested that the effect of green manure on soil fertility is limited by the relatively large amount of chemical fertilizer. The results reported herein can not only increase soil fertility and improve the soil ecological environment, but also enhance and stabilize the yields of double-season rice grown in the red paddy soil of southern China.

## 1. Introduction

Rice is the main source of nourishment for more than half of the Chinese population, so rice is considered an important food crop in China [1,2]. Rice is predominantly grown on the red soils of the southern region, particularly the Jiangxi Province. As a major rice-producing region in the south, the Jiangxi Province is ranked second and third in China in the rice cultivation area and total yield, respectively [2]. Of the 1.28 million km^2^ used to grow rice in southern China, 300,000 km^2^ (16%) are primarily composed of red paddy soil and produce 50% of China’s total rice yield [3,4]. Thus, the red paddy field is an important soil resource of China and plays a crucial role in maintaining food security [1,3,4].

China has abundant green manure resources, with more than 500 types of green manure and 3000 germplasms, it also has the world’s greatest green manure cultivation area of nearly 2 million hectares [5,6,7]. The Jiangxi Province is a traditional green manure-producing region, accounting for 20% of China’s green manure area, which is dominated by milk vetch-type manure, the Latin name of Chinese milk vetch is *Astragalus sinicus Linn* [8]. Under long-term non-fertilizer application conditions, the mean annual double-season rice yield in red and purple soil was about approximately 5–7 t·ha^−1^ [9,10,11,12,13,14]. The application of Chinese milk vetch with no chemical fertilizer had little effect on the mean annual double-season rice yields of yellow-brown and purple paddy soils. However, Chinese milk vetch combined with inorganic fertilizer raised the double-season rice production by approximately 80–100% in red and purple paddy soils [1,13,15,16]. Pig manure combining with NPK fertilizer and Chinese milk vetch raised the double-season rice production by approximately 90–110% in red paddy soil [1,11,12]. In general, the effect of long-term chemical fertilizer and green manure on the yield of double-season rice has been extensively studied. However, yield characteristics of paddy fields under different green manure cultivation modes are unclear.

There are many soil physic-chemical properties that affect rice yield, such as SOC, TN, TP, AK, pH, available nitrogen (AN) and available phosphorus (AP) [1,10,17,18]. However, these soil properties have different effects on rice yield [10,11,17,18]. Li et al. [16] found that AK, TP, SOC, AP, available zinc, and available manganese could explain 82.63% of the variation in rice yield under chemical fertilizer application, and the contribution of AK was the highest at 35.75%. Wu et al. [18] showed that AK, AP, and SOC can explain 99.74% of the variation in rice yield under the condition of no fertilizer and application of chemical fertilizer, among which AK contributed the most. Although there have been a lot of studies on the influence of soil physical and chemical properties on rice yield under the condition of no fertilizer and application of chemical fertilizer, the effect of soil properties on the rice yield under the long-term green manure fertilizer mode is still unknown.

Therefore, in order to provide a theoretical basis for high double-season rice yield and fertilizer application for red paddy soil, experiments were conducted under CK, NPK, NPK + GM, NPK + GM + S and NPK + GM + M application conditions. Our objectives were to (1) investigate the yield variation characteristics of double-season rice in the red paddy field under green manure cultivation modes and (2) determine the main factors affecting the yield of red paddy soil.

## 2. Materials and Methods

### 2.1. Experimental Site

The experiments were performed at the experimental base netted farm in Jiangxi Agricultural University (28°76′78″ N, 115°83′61″ E). The initial topsoil (0–20 cm) properties are shown in Table 1, soil texture is clay loam. The climate at this site is subtropical humid, with the mean annual temperature, mean annual precipitation, and sunshine hours being 17.1–17.8 °C, 1568–1655 mm, and 1772–1845 h. The site’s ≥ 0 °C cumulative temperature is 6256–6530 °C.

### 2.2. Experimental Design

The long-term experiment was started in 1981, and set up no fertilizer (CK), chemical fertilizer (NPK), NPK plus Chinese milk vetch (NPK + GM), NPK plus Chinese milk vetch and rice straw (NPK + GM + S), NPK plus Chinese milk vetch, and pig manure (NPK + GM + M). The experiments were performed in triplicates on 0.9 × 0.9 m^2^ micro-plots. The cropping system was double-season rice. The tested soil was derived from Quaternary red clay. This paper presents the rice yields and the soil’s physical-chemical characteristics determined within the period stretching from 1981 to 2009.

Early season rice was sown late in March, transplanted at the middle of April, then harvested at the begin of July. In total, 50 seedlings were grown in 25 stumps in every micro-plot area (2 seedlings per stump). The full growth season lasted around 100 days. As for late-season rice, it was sown at the end of June, transplanted at the end of July, and harvested in late October. Again, the seedlings in each micro-plot were grown in 25 stumps; however, each stump contained 4 seedlings. The full growth season lasted around 100 days.

### 2.3. Fertilizer Application

With the exception of CK, the four fertilization groups were all treated with the same amounts of chemical fertilizers. These chemical fertilizer amounts were increased as the study progressed (Table 2). The chemical fertilizers applied in our experiment included urea (N), calcium magnesium phosphate (P), and potassium chloride (K). The quantities of the chemical fertilizers were equally divided between the early and late seasons. Phosphorus fertilizer was applied in the seeding stage of rice only once, nitrogen and potassium fertilizer was applied in the seeding, tillering, and booting stage of rice in three times, and its ratio was 5:3:2, respectively.

The organic fertilizers are Chinese milk vetch, rice straw, and pig manure. Chinese milk vetch and rice straw was grown by the experimenter. Pig manure comes from surrounding farmer, and then composted by the experimenter. Fresh Chinese milk vetch is cut into small pieces to be used as early rice base fertilizer. The rice straws are cut finely and returned to the field, and the pig manure is returned to the field after composting. Rice straw and pig manure is the base fertilizer for late-season rice. The characteristics of Chinese milk vetch, rice straw, and pig manure are summarized in Table 3.

### 2.4. Rice Yield and Soil Sample Analysis

The rice crops were manually harvested during the maturation stage. After mechanical threshing, the rice was air-dried then weighed. The annual yields of different treatments were calculated by adding the quantities of rice produced in two seasons. The 0–20 cm soil samples were collected seven to ten days after the late rice harvest, and the sampling time is approximately from November 1st to 7th of each year. Five samples of the plow layer soil (0–20 cm) was collected from each plot, mixed into one composited sample. After collection, those samples were air-dried, ground and passed through 2 mm and 0.149 mm mesh sieves. The potassium dichromate volumetric method and Kjeldahl method was used to determine the SOC and TN, respectively. TP was determined by NaOH dissolution followed by molybdenum blue colorimetric assay, whereas TK was measured by NaOH dissolution followed by flame photometry. AN was measured using alkaline hydrolysis. AP was assessed via HCl-NH_4_F extraction followed by ammonium molybdate colorimetry, and AK was assessed via NH_4_OAC extraction followed by flame photometry. pH was determined using electrodes (water/soil ratio = 5:1) [19].

### 2.5. Data Analysis

One-way repeated ANOVA analyses were conducted using the SPSS Statistics 24 software (IBM, Armonk, NY, USA) in order to assess the variations between different treatment groups in terms of rice yield and the soil factors (*p <* 0.05). Meanwhile, the factor having significant effects on yield was identified by linear fitting, and the magnitudes of the effects of various factors were determined based on the redundancy analyses performed on Canoco 5. All graphs were plotted using Origin 8.5.

## 3. Results

### 3.1. Yield Variation Characteristics of Rice under Long-Term Green Manure Cultivation

**Figure 1:**
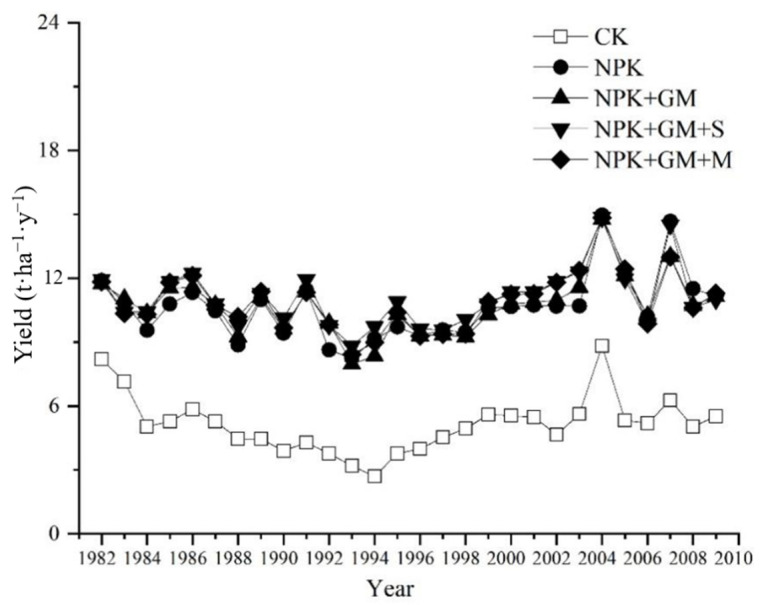
Profiles of temporal variation in double-season rice yield.

The annual rice yields in the five treatments showed similar trends of temporal variation, despite the fluctuations between different years. Between 1981 and 1994, the yields fluctuated slightly, but overall, a slowly decreasing trend was observed. The mean annual rice yields slowly raised between 1994 and 2004, and in the period of 2004–2009, they fluctuated greatly. The 2009 yields were found to be nearly the same as those recorded at the beginning of the study for all fertilizer treatments; however, the yield of the CK treatment had a decreased trend over the years (Figure 1).

**Figure 2:**
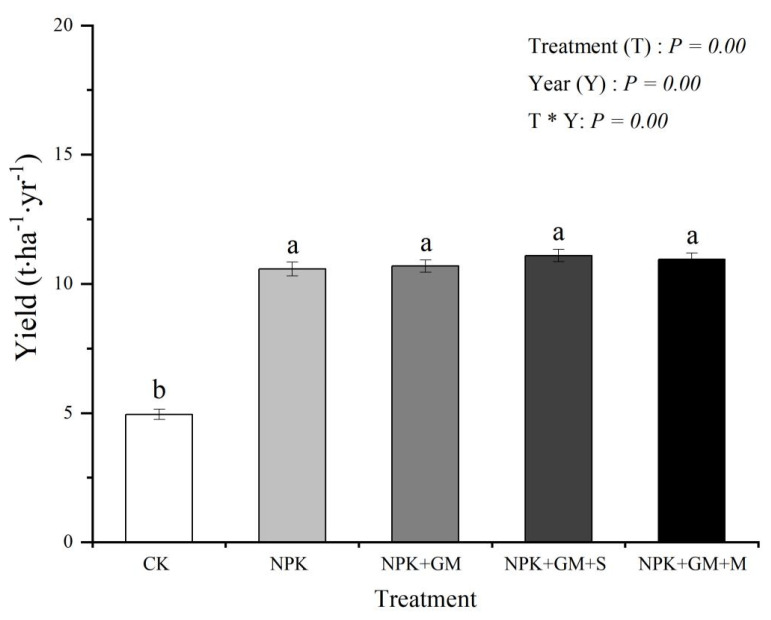
Effects of different treatments on rice yield.

The one-way repeated ANOVA results indicate that the treatment, year, and interaction of treatment × year all had a significant effect on double-season rice yield (*p* < 0.01) (Figure 2). Compared with the CK treatment, fertilization treatment can significantly raise the yield of rice, but there was no significance between NPK treatment and chemical fertilizer combined with green manure treatments (*p <* 0.05). The mean annual yield was 4.94 t·ha^−1^ under CK conditions and was 10.57–11.09 t·ha^−1^ for the NPK, NPK + GM, NPK + GM + S, and NPK + GM + M groups (Figure 2). Compared with the CK, NPK, NPK + GM, NPK + GM + S, and NPK + GM + M treatments increased mean annual rice yield by 113.97%, 116.40%, 124.49%, and 121.66%, respectively. This shows that chemical fertilizer combined with green manure can significantly increase the double-season rice yield of red paddy soil.

### 3.2. Factors Affecting Rice Yield

Correlation assessments of different soil properties and rice yield show that the yield was positively correlated with SOC, TN, TP, AN, AP, AK, and pH (*N* = 236, *p <* 0.01), and the correlation coefficients are 0.31, 0.37, 0.54, 0.22, 0.41, 0.57, and 0.55, respectively (Table 4).

### 3.3. Redundancy Analysis of the Effects of Soil Properties on Rice Yield

Redundancy analysis indicates that AK, pH, TN, TP, and SOC explained 32.2%, 15.3%, 10.5%, 5.1%, and 3.2% variation in rice yield, respectively (*p* < 0.05), for a total of 66.3% (Table 5). Therefore, AK, pH, TN, TP, and SOC may be considered as major factors affecting the double-season rice yield of red paddy soil.

**Figure 3:**
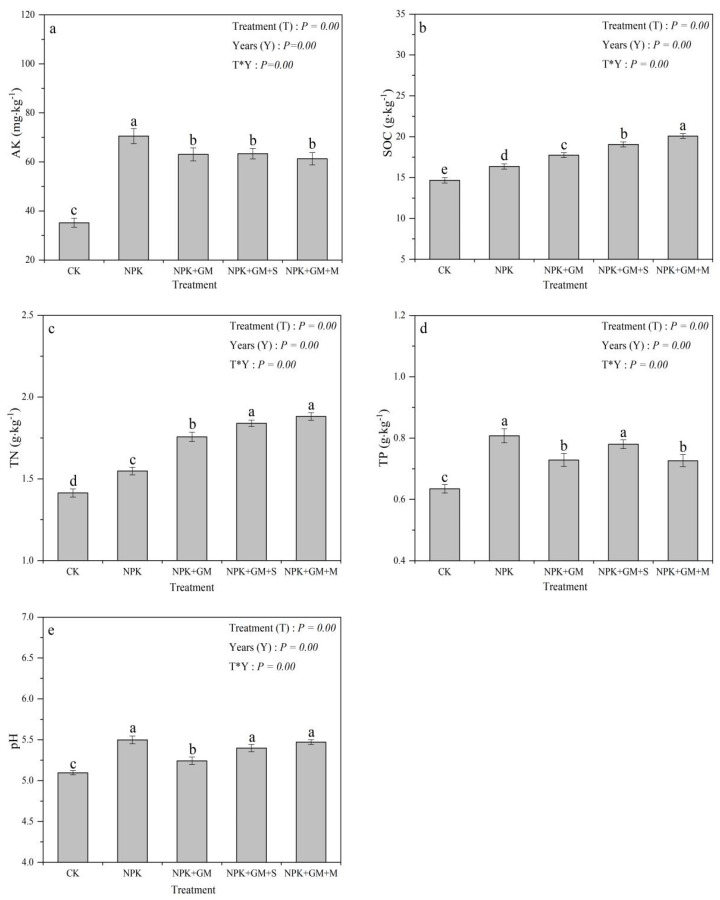
Effects of different treatments on the soil factors influencing the rice yield. The subfigure (**a**–**e**) represent the order of soil AK, SOC, TN, TP, pH, respectively. Different letters (**a**–**c**) indicates significant differences (*p* < 0.05) between treatments.

One-way repeated ANOVA showed that the fertilizer treatment, year, and the interaction of fertilizer treatment * year had extremely significant effects on soil AK, SOC, TN, TP, and pH (*p* < 0.01) (Figure 3). Compared with the CK treatment, four fertilizer treatments including NPK, NPK + GM, NPK + GM + S, and NPK + GM + M could significantly increase soil AK by 74.05–100.26%, among which the NPK treatment has the most significant effect; these fertilization treatments can significantly increase SOC by 11.53–36.97%, NPK+GM+M is the most significant treatment; fertilization treatments can significantly increase TN by 9.93–33.33%, among which NPK + GM + S and NPK + GM + M treatments has the best effect; fertilization treatments can significantly increase TP content by 15.87–22.22%, NPK and NPK + GM + S treatments has the best effect; fertilization treatments could significantly increase pH about 0.15–0.40 units, with NPK, NPK + GM + S and NPK + GM + M treatments having the most significant effects. Therefore, fertilization mainly increases rice yield by soil properties.

## 4. Discussion

### 4.1. Variation Characteristics of Double-Season Rice Yield under Long-Term Green Manure Cultivation

The results presented in this study clearly demonstrate that different modes of long-term green manure cultivation (NPK + GM, NPK + GM + S, and NPK + GM + M) could appreciably enhance the double-season rice yield of red paddy soil by 113.97%, 116.40%, 124.49%, and 121.66% (*p* < 0.01), respectively. This is consistent with several studies [12,13,20], they show that compared with the CK treatment, the NPK, NPK + GM, NPK + GM + S, or NPK + GM + M increased the mean annual rice yield by 39.19–80.30%. NPK + GM treatment increases the yield by promoting rice nutrition growth and augmenting the number of productive ears and panicles [14,21]. The addition of rice straw to NPK+GM further enhances rice production by increasing fertilizer utilization and providing more nutrients, particularly potassium, that may be readily absorbed and used by the rice [14,16,21]. NPK + GM + M improves the chemical characteristics and biotic environment of the soil, thereby facilitating crop growth and raising the yield [12,20].

Notably, the analyses performed herein indicate that there were no significant differences between the combined inorganic fertilizer plus Chinese milk vetch treatments and pure inorganic fertilizer (NPK) on yield. Several studies [3,12,14] reported similar conclusions, wherein they showed that combinations of chemical and organic fertilizers do not enhance rice yields more than chemical fertilizers alone. This may be attributed to the relatively high proportion of chemical fertilizers used in the combined treatments, which masks the benefits of organic fertilizers and undermines their persistent and slow-acting effects [12,22]. In our research, chemical fertilizer used in combination with green manure was increased over the years. Specifically, the amounts of N, P, and K fertilizers were increased from 220 to 300, from 112.5 to 250, and from 220 to 300 kg·ha^−^^1^, respectively (Table 2). Such excessive amounts of chemicals overpower the effects of organic fertilizers, which is the main reason for the lack of a significant difference between the yields of NPK and NPK + GM, NPK + GM + M, and NPK + GM + S groups. Consequently, we suggest the amount of inorganic fertilizer to cultivate rice in the red soils of southern China should be moderately reduced. It should be noted that some previous studies have clearly shown that combined fertilizers (chemical and organic) exhibit a superior effect in enhancing rice yields than pure chemical fertilizers [14,23]. Other research has found the difference in soil type may have some effect on yield, it could lead to no significance between the inorganic fertilizer plus organic fertilizer treatments [1,22,23]. This indicates that in addition to the amount of fertilizer, the soil type and characteristics are important factors affecting rice yield.

### 4.2. Effects of Soil Physical and Chemical Properties on Rice Yield

In our study, AK was the soil parameter that had the greatest influence on double-season rice yield as it accounted for 32.2% variation in yield. Meanwhile, one-way repeated ANOVA analyses indicated that there was significant difference in the AK levels among all treatments (Figure 3a), which indirectly demonstrates that AK is the key factor affecting crop yield. Other studies also show that AK can affect single-season rice, double-season rice, and wheat yields, with a contribution of up to 35.75% variance [15,18]. This may be due to the following reasons. First, red soil is deficient in potassium [18]. Second, decomposition of Chinese milk vetch can release a large amount of potassium [15,16], whereas rice straws contain large quantities of ionic potassium that can easily dissolve in water and enter the soil [1,14,16]. Third, potassium promotes photosynthesis and transportation of assimilated substances in rice, which ultimately favors crop growth and development [23].

TN explained a 10.5% variation in double-season rice yield, suggesting that TN is a main factor affecting double-season rice yield. One-way repeated measures ANOVA showed that all fertilizer treatment significantly increased the TN levels (Figure 3b), which indirectly demonstrates that TN is a factor affecting double-season rice yield. It has been reported that nitrogen has an important role in the crop growth cycle, so nitrogen could affect the rice yield [22,24]. Chinese milk vetch can improve soil nitrogen, and abundant nitrogen can significantly enhance the amount of effective panicle and the 1000 grain weight of rice crops. Concurrently, large amounts of nitrogen will significantly affect the physiological characteristics of crops, such as photosynthetic rate and chlorophyll content, promoting photosynthesis and accumulating the dry matter, thereby resulting in higher crop yield [22,24].

With a contribution of 5.1% variance, TP was another key factor affecting yield. All fertilizer treatments tested increased the TP contents in this study, albeit by varying degrees (Figure 3c), which further indicates that rice yields are affected by TP. Previously, studies [15] reported that TP could explain 15.14% of rice yield variance. Such great influence is attributed to the effect of combined fertilizer treatments in increasing acid phosphorus and rice root enzyme activities, which increases phosphorus content and activity and promotes phosphorus absorption and accumulation in rice [21,25,26,27]. Phosphorus also improves nutrient absorption and transportation and significantly enhances rice tillering, root system growth, ear nutrient accumulation, and plant dry weight, which ultimately increases the rice yield [23,28].

SOC explained 3.2% variation in rice yield, suggesting that SOC is a main factor affecting double-season rice yield. All fertilizer treatments significantly increased SOC levels (Figure 3d), which indirectly supports our conclusion. Many studies show a significant correlation between SOC and rice yield [22,29]. Adding Chinese milk vetch, straws, and pig manure introduces a lot of exogenous organic material into soil, with degradation by various enzymes and microorganisms, soil organic carbon content will significantly increase [22,30,31,32]. High levels of SOC promote soil stability and a uniform distribution of aggregate and SOC mineralization constitutes an essential source of mineral nutrients, which in turn are important to rice growth [31,32]. Increased SOC content enhances the soil’s biotic environment and fertility, thereby increasing the amount of available nutrients, which promotes normal growth and raises the crop yield [29,33].

Soil pH explained 15.3% variation in rice yield, suggesting that pH is a key factor influencing rice yield. Furthermore, one-way repeated ANOVA analyses indicated that the pH of all treatments was significantly different (Figure 3e). Studies have shown that pH is strongly correlated with yield, so it is a key limiting factor [34,35]. Decomposition of Chinese milk vetch plus straws or pig manure increases organic matter content in fields, and dissociation of functional groups (protonation of carbonyl groups) and release of basic cations from the organic matter increase soil pH [34,36,37]. At the same time, increasing acid soil pH can promote microbial activity and abundance, and elevate the content of available nutrient content [36,37]. Therefore, the pH can affect the double-season rice yield. When the soil pH is relatively low, it will cause adverse effects such as aluminum toxicity, reducing nutrient utilization, affecting the carbon and nitrogen cycle process, and reducing beneficial microorganisms. Improving acid soil pH is conducive to improving the soil ecological environment and food security.

## 5. Conclusions

The long-term green manure cultivation mode (NPK + GM, NPK + GM + M, and NPK + GM + S) can significantly increase the yield of double-season rice in red paddy soils, which facilitates stable and high rice yield. However, the amount of chemicals used should be moderately decreased. Soil properties AK, pH, SOC, TN, and TP are main factors affecting double-season rice yield in red paddy soil. Therefore, this study has a positive effect on improving the soil ecological environment and increasing rice yield, it not only clarified the factors affecting the yield of red paddy soil, but also provided a theoretical basis for stable and high yield of double-season rice and fertilization in the red soil region in China.

## Figures and Tables

**Table 1:** Initial physical and chemical properties of the 0–20 cm soil.

Soil Type	Soil Parent Material	pH	SOC (g·kg^−1^)	TN (g·kg^−1^)	AN (mg·kg^−1^)	AP (mg·kg^−1^)	AK (mg·kg^−1^)
Red soil	Quaternary red earth	6.5	18.9	1.8	90.0	20.8	87.0

**Table 2:** Amount of fertilizer applied yearly (kg·ha^−1^).

Treatment	Chinese Milk Vetch	Rice Straw.	Pig Manure	1981–1986 N-P_2_O_5_ -K_2_O	1987–1992 N-P_2_O_5_ -K_2_O	1993–2003 N-P_2_O_5_ -K_2_O	2004–2009 N-P_2_O_5_ -K_2_O
CK	0	0	0	0	0	0	0
NPK	0	0	0	225-112.5-225	240-120-240	270-135-270	300-150-300
NPK + GM	22,500	0	0	225-112.5-225	240-120-240	270-135-270	300-150-300
NPK + GM + S	22,500	3000	0	225-112.5-225	240-120-240	270-135-270	300-150-300
NPK + GM + M	22,500	0	15,000	225-112.5-225	240-120-240	270-135-270	300-150-300

**Table 3:** The characteristics of Chinese milk vetch, rice straw, and pig manure (fresh).

Fertilizer	N (%)	P (%)	K (%)
Chinese milk vetch	0.37–0.43	0.04–0.05	0.24–0.29
Rice straw	0.29–0.32	0.04–0.05	0.62–0.72
Pig manure	0.52–0.57	0.23–0.26	0.28–0.31

**Table 4:** Correlation between rice yield and influencing factors (*N* = 236).

	Yield	SOC	TN	TP	TK	AN	AP	AK	pH
Yield	1.00								
SOC	0.31 **	1.00							
TN	0.37 **	0.79 **	1.000						
TP	0.54 **	−0.34 **	−0.21 **	1.00					
TK	0.08	−0.63 **	−0.44 **	0.58 **	1.00				
AN	0.22 **	0.70 **	0.79 **	−0.20 **	−0.32 **	1.00			
AP	0.41 **	0.16 **	0.29 **	0.24 **	−0.04 **	0.24 **	1.00		
AK	0.57 **	0.16 **	0.24 **	0.31 **	0.02 **	0.12	0.45 **	1.00	
pH	0.55 **	−0.17 **	−0.09	0.77 **	0.36 **	−0.18	0.33 **	0.31 **	1.00

** *p* < 0.01.

**Table 5:** The redundancy analysis between rice yield and soil properties.

Indicator	Explains%	pseudo-F	P(adj)
AK	32.2	111	0.02 *
pH	15.3	67.8	0.02 *
TN	10.5	57.9	0.02 *
TP	5.1	32.2	0.02 *
SOC	3.2	21.6	0.02 *
Total	66.3		

P(adj), Significant value after adjustment. * means *p <* 0.05.

## Data Availability

No new data were created or analyzed in this study. Data sharing is not applicable to this article.
